# Driving Wheels and Strokes in a Dental Engine

**Published:** 1887-05

**Authors:** William Herbert Rollins


### DRIVING WHEELS AND STROKES IN A DENTAL ENGINE.

BY WILLIAM HERBERT ROLLINS.

The point on which I wish to speak is the importance of being able to vary the stroke and change the speed of a dental engine to suit the build of the person using the machine, and the particular work to be done. I shall confine my remarks to the Bonwill type of engine, as I should not care to use any other.

The pedal stroke in this engine is too long for the comfort of most operators. A tall man usually likes a long stroke, just as in walking he takes a long stride. For a short man the stroke should be shorter. The first point on which I wish to insist is, that the machines which a man uses should always be constructed to accord with his own structure. Unless this is done the amount of fatigue is unnecessarily increased.

The second point is, that in an engine with a high speed, you cannot adapt the speed to the work to be done as the engines are now made.

For example, with the strokes as rapid as is consistent with comfort, the speed is about right for the small burs ; but if we use a sandpaper disk of an ordinary size, say three quarters of an inch in diameter, it is almost impossible to run the machine slow enough to avoid unpleasant heating of the tooth and injurious expansion of the filling.

To modify the engine so that it shall not have these objections is a very simple matter, but one which the manufacturers have declined to do for me. My old engine is arranged to embody these changes in the following manner :

Turn off one side of the driving wheel till a flat surface is reached, and then fasten a smaller wheel, about eight inches in diameter onto this face.

Cut off the end of the hand-piece and extend the spindle enough to have a pully with a double groove.

Cut off the stud on the driving wheel, and in its place fasten to the wheel a bar of iron having a slot in which the stud can move. In this way the stroke can be varied from, say, four and a half inches to two inches, to suit the build of the operator. These changes are not expensive and are quickly done.

